# Diaqua­bis­{5-(pyridin-2-yl-κ*N*)-3-[4-(pyridin-4-yl)phen­yl]-1*H*-1,2,4-triazol-1-ido-κ*N* ^1^}cobalt(II)

**DOI:** 10.1107/S1600536813003243

**Published:** 2013-02-06

**Authors:** Bin Li

**Affiliations:** a Advanced Material Institute of Research, Department of Chemistry and Chemical Engineering, Qilu Normal University, Jinan 250013, People’s Republic of China

## Abstract

In the centrosymmetic title complex, [Co(C_18_H_12_N_5_)_2_(H_2_O)_2_], the Co^II^ ion is coordinated by two *N*,*N*′-bidentate 5-(pyridin-2-yl)-3-[4-(pyridin-4-yl)phen­yl]-1*H*-1,2,4-triazol-1-ide ligands and two water mol­ecules in a *trans*-CoO_2_N_4_ coordination geometry. In the ligand, the dihedral angles between the triazole ring and its adjacent pyridine and benzene rings are 5.57 (14) and 6.89 (16)°, respectively. In the crystal, mol­ecules are linked by O—H⋯N hydrogen bonds, generating a three-dimensional network.

## Related literature

For background to coordination complexes, see: Li *et al.* (2007 ▶); Zhang *et al.* (2012*a* ▶,*b* ▶); Fan *et al.* (2013 ▶).
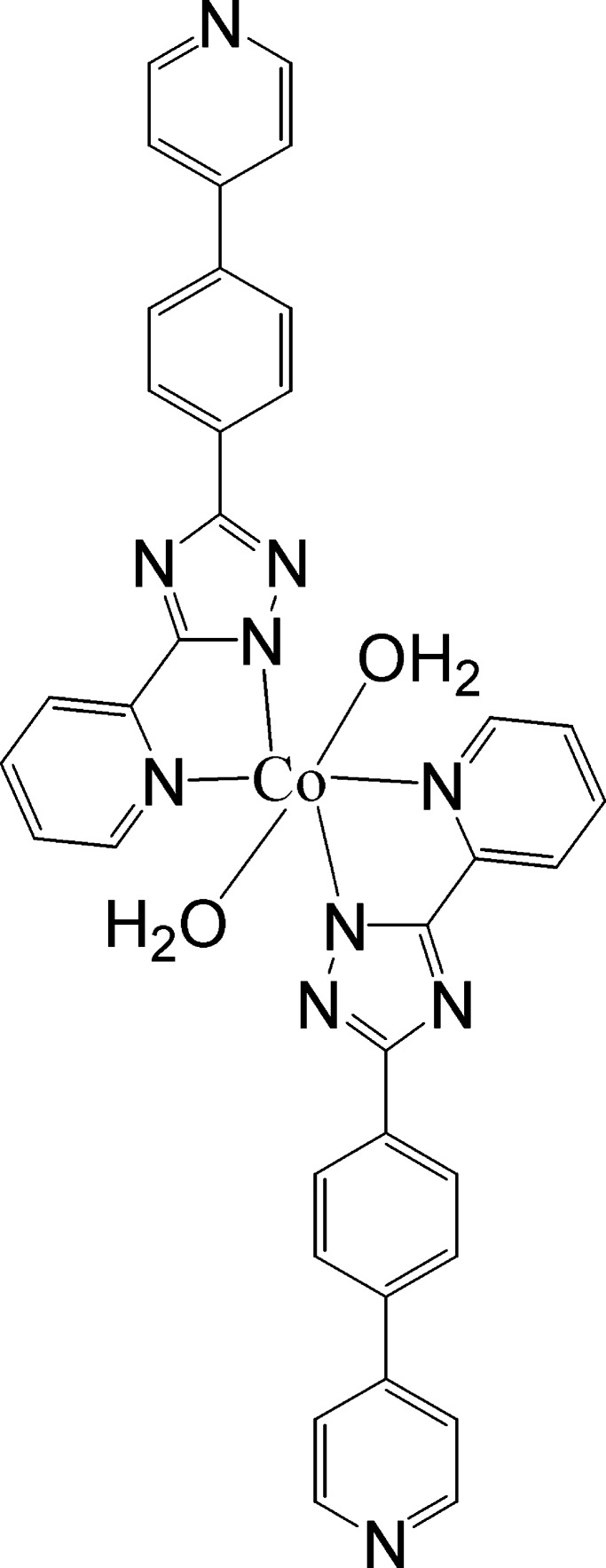


## Experimental

### 

#### Crystal data

[Co(C_18_H_12_N_5_)_2_(H_2_O)_2_]
*M* *_r_* = 691.61
Monoclinic, 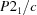

*a* = 13.2407 (17) Å
*b* = 11.9355 (16) Å
*c* = 9.8644 (13) Å
β = 101.158 (1)°
*V* = 1529.4 (3) Å^3^
*Z* = 2
Mo *K*α radiation
μ = 0.62 mm^−1^
*T* = 296 K
0.12 × 0.10 × 0.08 mm

#### Data collection

Bruker APEXII CCD diffractometer
Absorption correction: multi-scan (*SADABS*; Bruker, 2001 ▶) *T*_min_ = 0.930, *T*_max_ = 0.953
10448 measured reflections
2703 independent reflections
2227 reflections with *I* > 2σ(*I*)
*R*_int_ = 0.030

#### Refinement

*R*[*F* ^2^ > 2σ(*F* ^2^)] = 0.046
*wR*(*F* ^2^) = 0.139
*S* = 1.00
2703 reflections
229 parameters
3 restraints
H atoms treated by a mixture of independent and constrained refinement
Δρ_max_ = 1.05 e Å^−3^
Δρ_min_ = −0.39 e Å^−3^

### 

Data collection: *APEX2* (Bruker, 2004 ▶); cell refinement: *SAINT-Plus* (Bruker, 2001 ▶); data reduction: *SAINT-Plus*; program(s) used to solve structure: *SHELXS97* (Sheldrick, 2008 ▶); program(s) used to refine structure: *SHELXL97* (Sheldrick, 2008 ▶); molecular graphics: *SHELXTL* (Sheldrick, 2008 ▶); software used to prepare material for publication: *SHELXTL*.

## Supplementary Material

Crystal structure: contains datablock(s) global, I. DOI: 10.1107/S1600536813003243/hb7033sup1.cif

Structure factors: contains datablock(s) I. DOI: 10.1107/S1600536813003243/hb7033Isup2.hkl

Additional supplementary materials:  crystallographic information; 3D view; checkCIF report

## Figures and Tables

**Table 1:** Selected bond lengths (Å)

Co1—N2	2.057 (2)
Co1—N1	2.130 (2)
Co1—O1	2.181 (2)

**Table 2:** Hydrogen-bond geometry (Å, °)

*D*—H⋯*A*	*D*—H	H⋯*A*	*D*⋯*A*	*D*—H⋯*A*
O1—H1*W*⋯N4^i^	0.82 (1)	1.98 (1)	2.783 (3)	168 (4)
O1—H2*W*⋯N5^ii^	0.82 (1)	2.46 (3)	3.196 (4)	150 (5)

Symmetry codes: (i) 
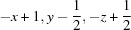
; (ii) 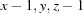.

**Fig. 1.**
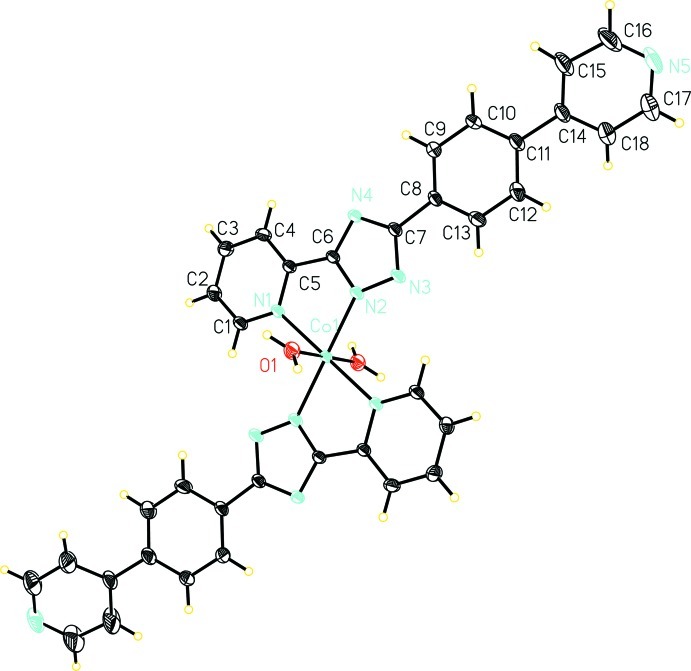
The title compound with displacement ellipsoids drawn at the 30% probability level. Unlabelled atoms are generated by the symmetry operation (1-x, -y, -z).

**Fig. 2.**
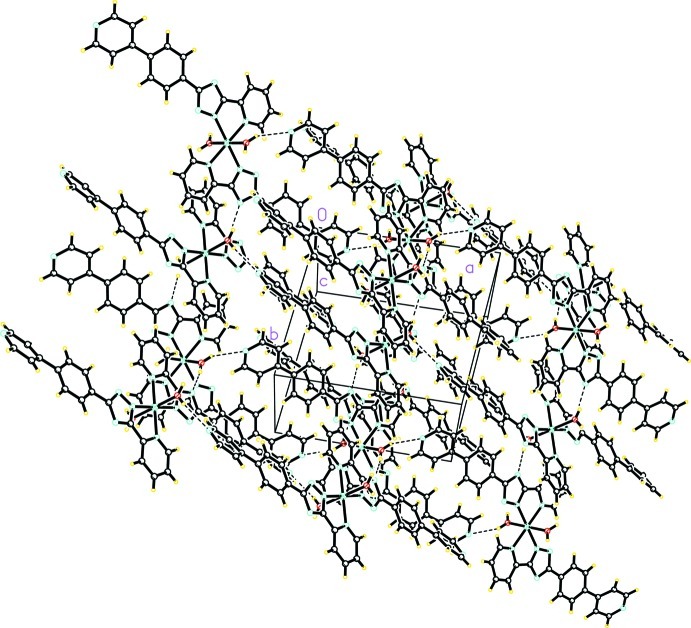
The crystal packing of the title compound, displayed with hydrogen bonds as dashed lines.

## supplementary crystallographic information

### Comment

The design and synthesis of coordination complexes have attracted upsurging research interest not only because of their appealing structural and topological novelty but also owing to their tremendous potential applications in gas storage, microelectronics, ion exchange, chemical separations, nonlinear optics and heterogeneous catalysis. (Li *et al.*, 2007; Zhang *et al.*, 2012**a*,b*; Fan *et al.*, 2013). Here, we report one new compound: Co(H_2_O)_2_(C_18_H_12_N_5_)_2_, obtained from the solvothermal reaction of 2-(3-(4-(pyridin-4- yl)phenyl)-1H-1,2,4-triazol-5-yl)pyridine and cobalt chloride.

The title compound, Co(H_2_O)_2_(C_18_H_12_N_5_)_2_, consists of of a half of Co(II), a half of depornated 2-(3-(4-(pyridin-4- yl)phenyl)-1H-1,2,4-triazol-5-yl)pyridine, and one associated water molecule. Co(1) owns a distorted octahedral coordination geometry, completed by four N atoms from two depornated 2-(3-(4-(pyridin-4- yl)phenyl)-1H-1,2,4-triazol-5-yl)pyridine and two O atoms from two water molecules (Figure 1). The Co—O distance is 2.181 (2) Å. The Co—N distances are varying from 2.057 (2)—2.130 (2) Å. O—H···N hydrogen bonding in the packing diagram leads to a consolidation of the structure (Fig. 2; Table 2) .

### Experimental

A mixture of 2-(3-(4-(pyridin-4- yl)phenyl)-1H-1,2,4-triazol-5-yl)pyridine (0.20 mmol, 0.060 g), cobalt(II) nitrate hexahydrate (0.40 mmol, 0.116 g), NaOH (0.20 mmol, 0.008 g) and 12 ml H_2_O was placed in a Teflon-lined stainless steel vessel, heated to 170 C for 3 days, followed by slow cooling (a descent rate of 10 C/h) to room temperature. Red blocks were obtained. Anal. Calc. for C_36_H_28_CoN_10_O_2_: C 65.52, H 4.08, N 20.25%; Found: C 65.45, H 4.02, N 20.22%.

### Refinement

All hydrogen atoms bound to carbon were refined using a riding model with distance C—H = 0.93 Å, *U*_iso_ = 1.2*U*_eq_ (C) for aromatic atoms. The H atoms of the water molecule were located from difference density maps and were refined with d(O—H) = 0.83 (2) Å, and with a fixed *U*_iso_ of 0.80 Å^2^.

### Crystal data

**Table:**

[Co(C_18_H_12_N_5_)_2_(H_2_O)_2_]	*F*(000) = 714
*M**_r_* = 691.61	*D*_x_ = 1.502 Mg m^−^^3^
Monoclinic, *P*2_1_/*c*	Mo *K*α radiation, λ = 0.71073 Å
Hall symbol: -P 2ybc	Cell parameters from 3600 reflections
*a* = 13.2407 (17) Å	θ = 2.3–26.5°
*b* = 11.9355 (16) Å	µ = 0.62 mm^−^^1^
*c* = 9.8644 (13) Å	*T* = 296 K
β = 101.158 (1)°	Block, red
*V* = 1529.4 (3) Å^3^	0.12 × 0.10 × 0.08 mm
*Z* = 2	

### Data collection

**Table:**

Bruker APEXII CCD diffractometer	2703 independent reflections
Radiation source: fine-focus sealed tube	2227 reflections with *I* > 2σ(*I*)
Graphite monochromator	*R*_int_ = 0.030
ω scans	θ_max_ = 25.0°, θ_min_ = 2.3°
Absorption correction: multi-scan (*SADABS*; Bruker, 2001)	*h* = −15→15
*T*_min_ = 0.930, *T*_max_ = 0.953	*k* = −13→14
10448 measured reflections	*l* = −11→11

### Refinement

**Table:**

Refinement on *F*^2^	Primary atom site location: structure-invariant direct methods
Least-squares matrix: full	Secondary atom site location: difference Fourier map
*R*[*F*^2^ > 2σ(*F*^2^)] = 0.046	Hydrogen site location: inferred from neighbouring sites
*wR*(*F*^2^) = 0.139	H atoms treated by a mixture of independent and constrained refinement
*S* = 1.00	*w* = 1/[σ^2^(*F*_o_^2^) + (0.084*P*)^2^ + 1.4448*P*] where *P* = (*F*_o_^2^ + 2*F*_c_^2^)/3
2703 reflections	(Δ/σ)_max_ = 0.016
229 parameters	Δρ_max_ = 1.05 e Å^−^^3^
3 restraints	Δρ_min_ = −0.39 e Å^−^^3^

### Special details

**Table:**

Geometry. All esds (except the esd in the dihedral angle between two l.s. planes) are estimated using the full covariance matrix. The cell esds are taken into account individually in the estimation of esds in distances, angles and torsion angles; correlations between esds in cell parameters are only used when they are defined by crystal symmetry. An approximate (isotropic) treatment of cell esds is used for estimating esds involving l.s. planes.
Refinement. Refinement of F^2^ against ALL reflections. The weighted R-factor wR and goodness of fit S are based on F^2^, conventional R-factors R are based on F, with F set to zero for negative F^2^. The threshold expression of F^2^ > 2sigma(F^2^) is used only for calculating R-factors(gt) etc. and is not relevant to the choice of reflections for refinement. R-factors based on F^2^ are statistically about twice as large as those based on F, and R- factors based on ALL data will be even larger.

### Fractional atomic coordinates and isotropic or equivalent isotropic displacement parameters (Å^2^)

**Table:**

	*x*	*y*	*z*	*U*_iso_*/*U*_eq_	
C1	0.3746 (2)	0.2081 (2)	−0.1267 (3)	0.0338 (7)	
H1	0.3430	0.1589	−0.1950	0.041*	
C2	0.3481 (2)	0.3190 (3)	−0.1381 (3)	0.0399 (7)	
H2	0.2998	0.3446	−0.2131	0.048*	
C3	0.3941 (3)	0.3920 (3)	−0.0371 (3)	0.0417 (8)	
H3	0.3774	0.4677	−0.0429	0.050*	
C4	0.4652 (2)	0.3519 (2)	0.0731 (3)	0.0364 (7)	
H4	0.4965	0.3999	0.1430	0.044*	
C5	0.4892 (2)	0.2395 (2)	0.0780 (3)	0.0269 (6)	
C6	0.5661 (2)	0.1858 (2)	0.1853 (3)	0.0272 (6)	
C7	0.6826 (2)	0.1420 (2)	0.3514 (3)	0.0297 (6)	
C8	0.7641 (2)	0.1417 (3)	0.4764 (3)	0.0338 (7)	
C9	0.7837 (2)	0.2303 (3)	0.5675 (3)	0.0433 (8)	
H9	0.7434	0.2946	0.5518	0.052*	
C10	0.8626 (3)	0.2249 (3)	0.6818 (3)	0.0478 (9)	
H10	0.8741	0.2858	0.7416	0.057*	
C11	0.9247 (2)	0.1313 (3)	0.7094 (3)	0.0421 (8)	
C12	0.9022 (3)	0.0412 (3)	0.6208 (4)	0.0563 (10)	
H12	0.9412	−0.0238	0.6380	0.068*	
C13	0.8234 (3)	0.0457 (3)	0.5078 (4)	0.0522 (9)	
H13	0.8095	−0.0169	0.4510	0.063*	
C14	1.0128 (2)	0.1248 (3)	0.8284 (3)	0.0467 (8)	
C15	1.0235 (4)	0.1889 (6)	0.9427 (5)	0.112 (2)	
H15	0.9742	0.2425	0.9511	0.134*	
C16	1.1092 (4)	0.1743 (7)	1.0485 (5)	0.120 (3)	
H16	1.1146	0.2213	1.1248	0.144*	
C17	1.1763 (4)	0.0477 (5)	0.9319 (5)	0.0846 (15)	
H17	1.2307	0.0008	0.9225	0.102*	
C18	1.0955 (3)	0.0567 (4)	0.8218 (5)	0.0771 (14)	
H18	1.0967	0.0162	0.7416	0.093*	
Co1	0.5000	0.0000	0.0000	0.0298 (2)	
N1	0.44390 (18)	0.16760 (19)	−0.0215 (2)	0.0289 (5)	
N2	0.58648 (18)	0.07828 (19)	0.1693 (2)	0.0320 (6)	
N3	0.66303 (19)	0.0488 (2)	0.2766 (2)	0.0349 (6)	
N4	0.62453 (18)	0.23101 (19)	0.2988 (2)	0.0292 (5)	
N5	1.1817 (3)	0.1015 (4)	1.0501 (3)	0.0734 (11)	
O1	0.38664 (16)	−0.04573 (19)	0.1239 (2)	0.0397 (5)	
H1W	0.374 (3)	−0.1106 (9)	0.143 (4)	0.080*	
H2W	0.343 (2)	0.000 (2)	0.137 (5)	0.080*	

### Atomic displacement parameters (Å^2^)

**Table:**

	*U*^11^	*U*^22^	*U*^33^	*U*^12^	*U*^13^	*U*^23^
C1	0.0342 (16)	0.0313 (15)	0.0302 (15)	−0.0007 (13)	−0.0078 (12)	−0.0025 (12)
C2	0.0414 (18)	0.0360 (17)	0.0355 (16)	0.0070 (14)	−0.0091 (13)	0.0047 (13)
C3	0.0509 (19)	0.0282 (16)	0.0403 (17)	0.0097 (14)	−0.0055 (15)	0.0020 (13)
C4	0.0434 (18)	0.0278 (15)	0.0326 (15)	−0.0004 (13)	−0.0061 (13)	−0.0049 (12)
C5	0.0279 (14)	0.0255 (14)	0.0247 (14)	−0.0014 (11)	−0.0013 (11)	−0.0008 (11)
C6	0.0281 (14)	0.0240 (14)	0.0263 (13)	−0.0018 (11)	−0.0031 (11)	−0.0011 (11)
C7	0.0290 (15)	0.0313 (15)	0.0249 (13)	−0.0009 (12)	−0.0040 (11)	−0.0001 (12)
C8	0.0297 (15)	0.0365 (16)	0.0299 (15)	0.0003 (13)	−0.0072 (12)	−0.0004 (13)
C9	0.0383 (18)	0.0457 (19)	0.0400 (17)	0.0101 (15)	−0.0072 (14)	−0.0099 (15)
C10	0.0400 (18)	0.058 (2)	0.0383 (17)	0.0087 (16)	−0.0099 (14)	−0.0203 (16)
C11	0.0351 (17)	0.057 (2)	0.0291 (15)	0.0027 (15)	−0.0069 (13)	−0.0035 (15)
C12	0.054 (2)	0.049 (2)	0.053 (2)	0.0133 (18)	−0.0222 (17)	−0.0011 (18)
C13	0.057 (2)	0.0392 (19)	0.048 (2)	0.0050 (17)	−0.0210 (17)	−0.0101 (16)
C14	0.0330 (17)	0.068 (2)	0.0334 (17)	0.0039 (16)	−0.0072 (14)	−0.0032 (16)
C15	0.068 (3)	0.191 (7)	0.059 (3)	0.050 (4)	−0.033 (2)	−0.052 (4)
C16	0.080 (4)	0.207 (8)	0.056 (3)	0.034 (4)	−0.030 (3)	−0.055 (4)
C17	0.058 (3)	0.108 (4)	0.075 (3)	0.013 (3)	−0.021 (2)	0.004 (3)
C18	0.060 (3)	0.094 (4)	0.065 (3)	0.023 (3)	−0.019 (2)	−0.012 (2)
Co1	0.0330 (3)	0.0221 (3)	0.0275 (3)	0.0006 (2)	−0.0110 (2)	−0.0016 (2)
N1	0.0292 (13)	0.0253 (12)	0.0272 (12)	−0.0005 (10)	−0.0066 (10)	−0.0006 (10)
N2	0.0336 (13)	0.0268 (13)	0.0291 (12)	0.0000 (10)	−0.0104 (10)	−0.0005 (10)
N3	0.0372 (14)	0.0288 (13)	0.0309 (13)	0.0006 (11)	−0.0133 (11)	−0.0008 (11)
N4	0.0304 (13)	0.0268 (13)	0.0263 (12)	−0.0010 (10)	−0.0043 (10)	−0.0035 (10)
N5	0.0438 (19)	0.121 (3)	0.0461 (19)	0.004 (2)	−0.0148 (15)	0.004 (2)
O1	0.0389 (12)	0.0321 (11)	0.0441 (12)	0.0015 (10)	−0.0019 (10)	0.0062 (11)

### Geometric parameters (Å, º)

**Table:**

C1—N1	1.336 (4)	C11—C14	1.488 (4)
C1—C2	1.368 (4)	C12—C13	1.372 (5)
C1—H1	0.9300	C12—H12	0.9300
C2—C3	1.374 (4)	C13—H13	0.9300
C2—H2	0.9300	C14—C15	1.348 (6)
C3—C4	1.379 (4)	C14—C18	1.375 (6)
C3—H3	0.9300	C15—C16	1.395 (6)
C4—C5	1.377 (4)	C15—H15	0.9300
C4—H4	0.9300	C16—N5	1.293 (7)
C5—N1	1.353 (3)	C16—H16	0.9300
C5—C6	1.466 (4)	C17—N5	1.321 (6)
C6—N2	1.328 (4)	C17—C18	1.374 (6)
C6—N4	1.344 (3)	C17—H17	0.9300
C7—N3	1.333 (4)	C18—H18	0.9300
C7—N4	1.354 (4)	Co1—N2^i^	2.057 (2)
C7—C8	1.473 (4)	Co1—N2	2.057 (2)
C8—C9	1.379 (4)	Co1—N1	2.130 (2)
C8—C13	1.391 (5)	Co1—N1^i^	2.130 (2)
C9—C10	1.381 (4)	Co1—O1^i^	2.181 (2)
C9—H9	0.9300	Co1—O1	2.181 (2)
C10—C11	1.383 (5)	N2—N3	1.363 (3)
C10—H10	0.9300	O1—H1W	0.8200 (11)
C11—C12	1.381 (5)	O1—H2W	0.8200 (11)
			
N1—C1—C2	122.8 (3)	C18—C14—C11	120.2 (3)
N1—C1—H1	118.6	C14—C15—C16	119.6 (5)
C2—C1—H1	118.6	C14—C15—H15	120.2
C1—C2—C3	118.8 (3)	C16—C15—H15	120.2
C1—C2—H2	120.6	N5—C16—C15	126.0 (5)
C3—C2—H2	120.6	N5—C16—H16	117.0
C2—C3—C4	119.4 (3)	C15—C16—H16	117.0
C2—C3—H3	120.3	N5—C17—C18	124.1 (5)
C4—C3—H3	120.3	N5—C17—H17	117.9
C5—C4—C3	118.9 (3)	C18—C17—H17	117.9
C5—C4—H4	120.5	C17—C18—C14	121.0 (4)
C3—C4—H4	120.5	C17—C18—H18	119.5
N1—C5—C4	121.7 (2)	C14—C18—H18	119.5
N1—C5—C6	113.3 (2)	N2^i^—Co1—N2	180.00 (19)
C4—C5—C6	125.0 (2)	N2^i^—Co1—N1	102.53 (9)
N2—C6—N4	112.9 (2)	N2—Co1—N1	77.47 (9)
N2—C6—C5	117.7 (2)	N2^i^—Co1—N1^i^	77.47 (9)
N4—C6—C5	129.3 (2)	N2—Co1—N1^i^	102.53 (9)
N3—C7—N4	114.0 (2)	N1—Co1—N1^i^	180.00 (12)
N3—C7—C8	119.6 (3)	N2^i^—Co1—O1^i^	89.65 (9)
N4—C7—C8	126.3 (3)	N2—Co1—O1^i^	90.35 (9)
C9—C8—C13	117.4 (3)	N1—Co1—O1^i^	88.49 (9)
C9—C8—C7	124.0 (3)	N1^i^—Co1—O1^i^	91.51 (9)
C13—C8—C7	118.6 (3)	N2^i^—Co1—O1	90.35 (9)
C8—C9—C10	120.9 (3)	N2—Co1—O1	89.65 (9)
C8—C9—H9	119.6	N1—Co1—O1	91.51 (9)
C10—C9—H9	119.6	N1^i^—Co1—O1	88.49 (9)
C9—C10—C11	121.7 (3)	O1^i^—Co1—O1	180.00 (15)
C9—C10—H10	119.2	C1—N1—C5	118.4 (2)
C11—C10—H10	119.2	C1—N1—Co1	126.26 (19)
C12—C11—C10	117.2 (3)	C5—N1—Co1	115.22 (18)
C12—C11—C14	119.9 (3)	C6—N2—N3	107.2 (2)
C10—C11—C14	122.9 (3)	C6—N2—Co1	116.21 (18)
C13—C12—C11	121.4 (3)	N3—N2—Co1	136.62 (19)
C13—C12—H12	119.3	C7—N3—N2	104.5 (2)
C11—C12—H12	119.3	C6—N4—C7	101.4 (2)
C12—C13—C8	121.4 (3)	C16—N5—C17	113.7 (4)
C12—C13—H13	119.3	Co1—O1—H1W	124 (2)
C8—C13—H13	119.3	Co1—O1—H2W	120 (2)
C15—C14—C18	114.8 (4)	H1W—O1—H2W	114.6 (2)
C15—C14—C11	124.8 (4)		

Symmetry code: (i) −*x*+1, −*y*, −*z*.

### Hydrogen-bond geometry (Å, º)

**Table:**

*D*—H···*A*	*D*—H	H···*A*	*D*···*A*	*D*—H···*A*
O1—H1*W*···N4^ii^	0.82 (1)	1.98 (1)	2.783 (3)	168 (4)
O1—H2*W*···N5^iii^	0.82 (1)	2.46 (3)	3.196 (4)	150 (5)

Symmetry codes: (ii) −*x*+1, *y*−1/2, −*z*+1/2; (iii) *x*−1, *y*, *z*−1.
